# The hidden dancers in water: the symbiotic mystery of *Legionella pneumophila* and free-living amoebae

**DOI:** 10.3389/fmicb.2025.1634806

**Published:** 2025-08-08

**Authors:** Yuehua Wang, Linzhe Jiang, Fei Zhou, Yi Zhang, Ryan D. Fine, Mingguang Li

**Affiliations:** ^1^School of Laboratory Medicine, Jilin Medical University, Jilin, China; ^2^General Surgery, Jilin People’s Hospital, Jilin, China; ^3^Medical Image Center, Jilin Central Hospital, Jilin, China; ^4^Department of Biomedical Sciences, Cedars-Sinai Medical Center, Los Angeles, CA, United States; ^5^Center for Human Genetics and Genomics, New York University Grossman School of Medicine, New York, NY, United States

**Keywords:** *Legionella pneumophila*, free-living amoebae, symbiotic relationship, pathogenicity, public health

## Abstract

*Legionella pneumophila*, a Gram-negative bacillus, is the primary etiological agent of Legionnaires’ disease, a severe respiratory infection. The symbiotic relationship between *L. pneumophila* and free-living amoebae (FLAs), particularly *Acanthamoeba* spp., represents a critical intersection of microbial ecology and human pathogenesis. This symbiosis provides *Legionella* with a protective intracellular niche, enhancing its resistance to biocides, increasing its pathogenicity, and facilitating horizontal gene transfer. These interactions not only boost the environmental persistence and dissemination of *L. pneumophila* but also elevate the risk of human exposure through contaminated drinking water systems. This review delves into the sophisticated survival strategies employed by *L. pneumophila* within host cells, including evasion of endocytic pathways, inhibition of phagosome maturation and acidification, and prevention of phagosome-lysosome fusion. By elucidating these mechanisms, we underscore the critical need for in-depth research into the *Legionella-*amoebae symbiosis and its broader implications for public health. Additionally, we address the challenges and strategies for mitigating environmental risks, emphasizing the importance of innovative approaches to ensure water system safety and prevent pathogen transmission.

## 1 Introduction

Symbiosis exemplifies coexistence and coevolutionary trajectories across diverse life forms. These interactions, particularly between prokaryotes and environmental protists, are predominantly driven by nutritional exchanges with three main classes: mutualistic, commensalistic, and parasitic. However, the seminal work of Lynn Margulis in 1967 proposed the sophisticated endosymbiotic theory of organelle origins for mitochondria and chloroplasts (Sagan, 1967), which catalyzed a paradigm shift in understanding symbiotic relationships between unicellular eukaryotes and prokaryotes. Endosymbiosis, where one organism resides within another, is prevalent between protists and bacteria, underpinning adaptive evolution across multiple biological lineages (Nowack and Melkonian, 2010; Wernegreen, 2012). Such relationships facilitate niche adaptation and ecological diversification. Notably, free-living amoebae (FLAs) have emerged as critical hosts for a spectrum of microorganisms, including bacteria, viruses, and even other eukaryotes. These interactions confer advantages such as predation resistance, ecological resilience, and enhanced intracellular survival and proliferation.

The evolutionary interplay between protists and bacteria has been a cornerstone of research, with horizontal gene transfer from bacteria to protist hosts profoundly shaping protist genomes (López-García et al., 2017). While the evolutionary significance of prokaryote-eukaryote endosymbiosis is well-documented, its implications for human health remain underexplored. A quintessential example of such symbiosis is the relationship between *Legionella pneumophila*, a respiratory pathogen, and FLAs, a group of opportunistic pathogens. Their symbiosis is characterized by widespread environmental distribution from natural water sources to man-made water system with unique lifestyle adaptations befitting such extremes, which underscores the intricate dynamics of their unicellular eukaryote-bacteria interactions.

### 1.1 L. pneumophila

*L. pneumophila* belongs to the genus *Legionella* and is the primary pathogen responsible for severe pulmonary inflammation known as Legionnaires’ disease. It is also the most common pathogenic species within this genus. As a Gram-negative bacillus, *L. pneumophila* is characterized by its facultative intracellular parasitism (Cunha et al., 2016). This bacterium is widely distributed in nature and can be isolated from both soil and various aquatic environments. Notably, freshwater systems (Zhan and Zhu, 2018; Fliermans, 1996; van Heijnsbergen et al., 2015) and man-made water supply systems (van Heijnsbergen et al., 2015; Colbourne and Dennis, 1985; Wadowsky et al., 1982) often serve as its primary habitats. *L. pneumophila* exhibits remarkable adaptability, capable of surviving within a broad temperature range of 6°C to 63°C (Fliermans et al., 1981). Research has shown that the high-temperature environment of hot springs provides ideal conditions for its growth (Hsu et al., 2011; Ji et al., 2014; Rasch et al., 2016). This finding may explain why, compared to natural water systems, man-made water systems, with their higher average water temperatures (Ikedo and Yabuuchi, 1986; Fields et al., 2002; Lasheras et al., 2006), tend to support a greater abundance of these bacteria (Yamamoto et al., 1992).

*L. pneumophila* can exist as planktonic cells but predominantly colonizes biofilms under fluctuating and nutrient-poor conditions to acquire essential nutrients for survival and growth (Mampel et al., 2006; Singh and Coogan, 2005; Declerck et al., 2009; Declerck et al., 2007b). This pathogen integrates into existing biofilm structures (Lau and Ashbolt, 2009; Stewart et al., 2012) and establishes cooperative relationships with other biofilm-associated bacteria and cyanobacteria to enhance nutrient acquisition (Stewart et al., 2012; Tison et al., 1980; Bohach and Snyder, 1983; Stout et al., 1986; Koide et al., 2014; Pope et al., 1982). Remarkably, *L. pneumophila* employs a unique survival strategy by utilizing breakdown products from dead cell aggregates to sustain its metabolic activities in nutrient-scarce environments (Temmerman et al., 2006). While interactions with other bacteria facilitate survival in nutrient-deficient settings, proliferation within protozoan hosts remains a primary mode of its replication in natural habitats (Rowbotham, 1980).

The discovery of *L. pneumophila* traces back to the 1976 outbreak of Legionnaires’ disease among veterans attending a convention in Philadelphia, USA. Over 200 attendees fell ill, and 29 died, including bystanders outside the hotel (Fraser et al., 1977). Patients exhibited pneumonia-like symptoms, such as cough, headache, and high fever, with severe cases requiring prolonged hospitalization. This outbreak underscored the potential of water systems as reservoirs for respiratory pathogens (Fraser et al., 1977; McDade et al., 1977). Subsequent studies confirmed that infection occurs through inhalation of contaminated aerosols, with *L. pneumophila* invading lung macrophages and replicating intracellularly, leading to pneumonia (Steinert et al., 2007). Approximately 90% of Legionaries’ disease infections are attributed to *L. pneumophila* (Miyashita et al., 2020), with immunocompromised and elderly populations at heightened risk of severe outcomes, including respiratory failure (Fields et al., 2002; Gomez-Valero et al., 2009). In contrast, healthy individuals typically mount effective innate immune responses to control infection (Falcó et al., 1991; Shin, 2012).

Legionnaires’ disease exhibits seasonal peaks during summer and fall and is transmitted exclusively through airborne routes, with no evidence of human-to-human transmission (Burillo et al., 2017). Clinically, it presents in two forms: Pontiac fever, a mild flu-like illness that resolves spontaneously, and severe atypical pneumonia, characterized by acute respiratory symptoms, multi-organ damage, and high mortality if left untreated (Cunha et al., 2016). The incidence of Legionnaires’ disease has risen globally, with reported cases in the United States increasing ninefold from 2000 to 2018 (Yu et al., 2024), and doubling in Europe between 2010 and 2019 (Network, The European Centre for Disease Prevention and Control, 2021). Approximately 10–25% of cases result in fatalities, particularly among the elderly and immunocompromised individuals (World Health Organization, 2016). Healthcare-associated outbreaks are notably severe, with mortality rates reaching as high as 50% (Gomez-Valero et al., 2011; Lin et al., 2011; Marrie, 2008; Blatt et al., 1993; Sabria and Yu, 2002). Overall, mortality rates range from 10 to 25% (Yu et al., 2024), with higher rates observed in high-risk populations such as immunocompromised patients (Phin et al., 2014).

### 1.2 FLAs

FLAs are a diverse group of heterotrophic protists ubiquitously distributed in natural environments. Taxonomically, they span multiple phylogenetic lineages (Otero-Ruiz et al., 2022; Pazoki et al., 2020). Pathogenic genera affecting humans and animals include *Acanthamoeba* spp., *Balamuthia mandrillaris*, *Naegleria fowleri*, and *Sappinia pedata* (Schuster and Visvesvara, 2004; Visvesvara et al., 2007), with *Vermamoeba vermiformis* also recognized as an emerging pathogenic free-living amoeba (FLA) capable of causing human infections (Scheid, 2019). Notably, *Dictyostelium discoideum* exhibits typical FLA characteristics (e.g., trophozoite morphology and phagocytic activity) while serving as a unique ‘social amoeba’ model. Its distinctive life cycle, which transitions from unicellular individuals to multicellular fruiting bodies under starvation, makes it invaluable for studying cell differentiation, signal transduction, and host-pathogen interactions (Bozzaro et al., 2019; Swart et al., 2018).

FLAs share a biphasic life cycle: (1) a metabolically active *trophozoite* stage that feeds, divides, and moves via pseudopodia; and (2) a dormant *cyst* stage resistant to environmental stressors such as nutrient deprivation, extreme pH, osmotic fluctuations, and temperature variations (Marciano-Cabral, 2009; Bellini et al., 2022). These organisms pose significant public health risks through both opportunistic and non-opportunistic infections. Central nervous system (CNS) infections include primary amoebic meningoencephalitis (PAM), an acute condition caused by *N. fowleri* (Güémez and García, 2021; Gharpure et al., 2021), and granulomatous amoebic encephalitis (GAE), a subacute-to-chronic disease induced by *Acanthamoeba* spp. and *B. mandrillaris* (Spottiswoode et al., 2024; Lee et al., 2020). The latter two pathogens are also implicated in cutaneous lesions and disseminated infections, primarily affecting immunocompromised individuals (Cabello-Vílchez and Ruiz-Ruiz, 2024; Maehara et al., 2022). Ocular infections include *Acanthamoeba* keratitis (AK), a progressive sight-threatening corneal disease (Garg and Daigavane, 2024; Petrillo et al., 2024), while *S. pedata* and *V. vermiformis* are rarely reported in encephalitis and keratitis cases, respectively (da Rocha-Azevedo et al., 2009; Qvarnstrom et al., 2009; Scheid, 2019).

Beyond direct pathogenicity, FLAs play critical ecological roles as microbial predators, shaping aquatic and soil microbiota through bacterivory (Fan et al., 2024; Avery et al., 1995; Santos, 2025). Their phagocytic activity facilitates the intracellular harboring of bacteria, fungi, viruses, and other microbes, earning them the designation “Trojan horses” for microbial transmission (Scheid, 2014; Rayamajhee et al., 2021; Barker and Brown, 1994). FLAs thrive in diverse habitats ranging from natural systems (soil, freshwater, marine environments) (Rodríguez-Zaragoza, 1994; Tawfeek et al., 2016) to artificial niches (biofilms, anthropogenic water systems including wastewater plants, cooling towers, and swimming pools) (Puzon et al., 2009; Üstüntürk-Onan and Walochnik, 2018; Shaheen et al., 2019; Pagnier et al., 2009; Potgieter et al., 2021; da Silva et al., 2024). Their prevalence in nutrient-rich sediments and biofilms (Potgieter et al., 2021; da Silva et al., 2024), coupled with expanding artificial water infrastructure, heightens human exposure to both FLAs and their symbiotic pathogens, escalating potential zoonotic risks.

### 1.3 Discovery of the symbiotic phenomenon between *Legionella pneumophila* and FLAs

The concept of bacteria as intracellular symbionts in amoebae was first recognized in 1975 (Proca-Ciobanu et al., 1975), with *Acanthamoeba* identified as a host for pathogenic microorganisms in 1978 (Krishna Prasad and Gupta, 1978). Early studies in 1967 (Jeon and Lorch, 1967) and 1975 (Proca-Ciobanu et al., 1975) observed bacteria within *Acanthamoeba castellanii* cells. Over the following decades, Jeon and Jeon (1976) published a series of studies detailing the infection of amoebae by an endogenous affinity bacterium (termed X-bacterium), later identified as *L. jeonii* (Park et al., 2004). Rowbotham (1980) first reported that *L. pneumophila* could survive and replicate within *Acanthamoeba* and *Naegleria*. Further studies revealed that *L. pneumophila* thrives in drinking water only in the presence of amoebae (Wadowsky et al., 1988) and can remain viable for up to 6 months in *Acanthamoeba*-containing cultures (Bouyer et al., 2007), whereas free-living *Legionella* in biofilms may lose viability within weeks (Declerck et al., 2009; Murga et al., 2001). These findings have spurred significant interest in the interactions between FLAs and pathogenic bacteria, particularly the intricate relationship between *L. pneumophila* and *Acanthamoeba* spp., which has emerged as a major research focus. The reported strains of *L. pneumophila* that are capable of engaging in symbiosis with amoebae are shown in Supplementary Table 1.

Earlier sequencing studies revealed that the genome of *A. castellanii* (Neff) contains approximately 15,450 intron-rich genes, many of which are thought to originate from extensive horizontal gene transfer between *A. castellanii* and bacteria, archaea, viruses, and other eukaryotes (Clarke et al., 2013). Notably, most of these genes are integrated into *A. castellanii*’s transcriptional program and are actively expressed (Clarke et al., 2013). Compared to other species, the genomes of several bacteria infecting *Acanthamoeba* are significantly larger (Moliner et al., 2010). Researchers have identified numerous horizontally transferred genes and mobile genetic elements, such as integrases and transposases, in the genomes of intracellular bacteria, including *L. pneumophila*, *L. drancourtii*, *Candidatus “Protochlamydia amoebophila*,” *Rickettsia bellii*, and *Candidatus “Amoebophilus asiaticus*.” A large number of insertion sequences have also been detected in the *L. pneumophila* genome (Moliner et al., 2010). Further studies reveal distinct gene expression patterns in *L. pneumophila* grown in human monocytes versus *Acanthamoeba* (Mou and Leung, 2018), suggesting host-specific adaptation. Certain adaptive changes may be essential for survival in *Acanthamoeba* but unnecessary in macrophages, and vice versa. In this review, we primarily examine the symbiotic mechanisms between *Acanthamoeba* spp. and *L. pneumophila*, while secondarily addressing interactions involving other FLAs and *Legionella* species. We evaluate how these relationships collectively impact human health, underscoring the critical role of symbiosis in microbial pathogenesis and its public health implications.

## 2 Symbiotic mechanisms of *Legionella pneumophila* and FLAs

### 2.1 The first step of symbiosis—adhesion

Adhesion of *L. pneumophila* to host cells is the primary step in establishing its ecological niche and a critical factor for successful symbiosis. This includes formation of biofilms that are highly resistant to environmental exposure and increase long term resilience. Key bacterial factors involved in *L. pneumophila* adhesion to *Acanthamoeba* surfaces include RtxA cytotoxin, *Legionella* collagen-like protein (Lcl), pillins such as PilE, the lipopolysaccharide (LPS), the putative *L. pneumophila*-specific adenylate cyclase (LadC) and the periplasmic protein, EnhC. The majority of these factors have evolved to target host cell membrane receptor proteins and sugars for binding, which enables mechanical attachment to facilitate invasion.

Although its precise mechanism remains incompletely understood, RtxA is thought to facilitate the adhesion and invasion of *A. castellanii* by binding to β2 integrin-like receptors (Ambagala et al., 1999; Lally et al., 1997). However, such receptors have not been identified in *Acanthamoeba* to date. Integrin-like receptors have been found in other amoebae, such as *Hartmannella* (Venkataraman et al., 1997) and *Entamoeba* (Adams et al., 1993; Vines et al., 1998), which share immunoreactive epitopes with human β2 integrins (Adams et al., 1993) and contain characteristic β2 integrin motifs in their cytoplasmic domains (Vines et al., 1998). This suggests that similar receptors may exist in macrophages and amoebic trophozoites, potentially mediating *L. pneumophila* binding via RtxA. Notably, the *rtxA* gene is not universally present in all *Legionella* species, and its presence correlates closely with their ability to cause human disease (Cirillo et al., 2002; Cirillo et al., 2001). Thus, further investigation into RTX protein-mediated adhesion and entry mechanisms could reveal novel therapeutic targets for *Legionella* infections.

Lcl is an extracellular membrane protein that recognizes sulfated glycosaminoglycans (GAGs) on eukaryotic cell surfaces and promotes bacterial aggregation in the presence of divalent cations (Rehman et al., 2024). GAGs are diverse linear carbohydrates composed of repeating disaccharide units of amino sugars (N-acetylglucosamine or N-acetylgalactosamine) and glucuronic acid or galactose (Gandhi and Mancera, 2008). These complex carbohydrates play crucial roles in various biological processes, including cell adhesion, cell signaling, and microbial pathogenesis (Gandhi and Mancera, 2008). The C-terminal domain of Lcl (Lcl-CTD) is highly conserved (>97%) across *L. pneumophila* strains and forms a unique trimer structure with a deep negatively charged cavity and a positively charged external surface (Rehman et al., 2024). The positively charged surface of Lcl-CTD is critical for interacting with the negatively charged sulfate groups of GAGs, enabling Lcl to mediate bacterial adhesion to host cells and biofilm formation (Rehman et al., 2024). Lcl plays a significant role in the biofilm formation of *L. pneumophila*. Biofilms are structures composed of a polysaccharide matrix secreted by bacteria, which protect the bacteria from external environmental stresses and promote bacterial colonization and infection in host tissues (Abdel-Nour et al., 2019). Studies demonstrate that Lcl expression is essential for *L. pneumophila* aggregation and biofilm stability and mutations in the *lcl* gene severely impair bacterial adhesion to biofilms (Chatfield et al., 2020; Abdel-Nour et al., 2019). Additionally, the N-terminal region of Lcl anchors the protein to the bacterial surface, localizing it to the outer membrane. This anchoring mechanism likely stabilizes Lcl’s surface display, enhancing its interaction with host cell GAGs (Rehman et al., 2024). Collectively, Lcl’s structural and functional properties establish it as a key virulence factor in *L. pneumophila* infections and a potential target for novel antibacterial strategies.

The *PilE* gene encodes type IV pili in *L. pneumophila*, which are essential for adhesion to *A. polyphaga* and DNA transformation (Stone and Abu Kwaik, 1998; Stone and Kwaik, 1999). As a major structural component, PilE facilitates pili assembly, extension, and retraction (Nguyen et al., 2015), enabling attachment to both protozoan hosts and human cells, a dual role critical for environmental survival and pathogenicity (Stone and Abu Kwaik, 1998). PilE was initially identified through a genetic screen for adherence-deficient mutants, highlighting its importance in early infection (Stone and Abu Kwaik, 1998). While PilE is a key virulence factor, RtxA which was mentioned earlier, also contributes to adhesion. Notably, *PilE*- and *RtxA*-deficient mutants exhibit significantly reduced adhesion and invasion in epithelial cells and monocytes (Stone and Abu Kwaik, 1998; Cirillo et al., 2000), suggesting complementary roles via distinct host receptors. PilY1, a non-pilin protein associated with type IV pili, localizes at the pilus tip and functions as an adhesin for diverse substrates (Guo et al., 2024). It regulates pili dynamics, including calcium-dependent twitching motility and mechanosensing (Guo et al., 2024). During the stationary phase (a high-virulence state), PilY1 surface expression promotes *L. pneumophila* infection of human lung explants by enhancing adhesion, invasion, and motility (Hoppe et al., 2017). Specifically, PilY1 mediates adherence to alveolar epithelial cells (A549) and macrophages (THP-1), while its C-terminal PilY domain restores wild-type adherence, and the vWFa domain facilitates invasion into non-phagocytic cells (Hoppe et al., 2017). These findings implicate PilY1 in breaching epithelial barriers through multifunctional mechanisms. Given the conserved adhesion strategies between amoebae and macrophages, further studies should clarify the precise roles of PilE and PilY1 in *L. pneumophila*’s interaction with FLAs.

LPS composition of the bacterial outer membrane is a key determinant of *L. pneumophila*’s ability to adhere to host cells (Palusinska-Szysz et al., 2019). Studies have shown that LPS structure significantly influences the bacterium’s interaction with amoebal hosts such as *A. castellanii*. For instance, the wild-type Corby strain, possessing full-length LPS, exhibits highly efficient and rapid binding to the amoeba surface, followed by successful host cell invasion. In contrast, the TF3/1 mutant, which lacks high-molecular-weight LPS fractions, demonstrates reduced adhesion efficiency and impaired internalization, underscoring the essential role of LPS in mediating initial contact and subsequent host cell entry (Palusinska-Szysz et al., 2019). Among the 72 known Legionella species, *L. pneumophila* is the primary pathogen, responsible for 80–90% of legionellosis cases in Europe and the United States (European Centre for Disease Prevention and Control, 2021). This species can be classified into 15 serogroups based on conventional serotyping. Although serogroups 2–15 (Sg 2–15) are frequently detected in healthcare facilities, Sg 1 accounts for approximately 90% of clinical cases (Chahin and Opal, 2017; Alexandropoulou et al., 2015). Supplementary Table 2 summarizes the epidemiological characteristics, environmental distribution, and control strategies corresponding to each serogroup of *L. pneumophila*. The LPS of *L. pneumophila* Sg 1 consists of three major components: a surface-exposed O-specific chain, a core oligosaccharide, and lipid A. The core region contains sugars such as rhamnose, mannose, acetylquinovosamine, and acetylglucosamine, which facilitate interactions with eukaryotic cell surface receptors. These interactions are critical for the initial adhesion of *L. pneumophila* to *A. castellanii* (Kowalczyk et al., 2023). Interestingly, a mutant strain lacking O-acetyl groups on the rhamnose residues of the LPS core region exhibited enhanced adhesion to *A. castellanii* compared to the wild-type strain (Kowalczyk et al., 2023). This suggests that O-acetylation modulates host-pathogen interactions, possibly by altering bacterial surface properties and improving compatibility with amoebal receptors. Furthermore, the O-acetyl-deficient mutant displayed higher intracellular replication efficiency, indicating that LPS structure not only governs initial adhesion but also influences bacterial survival and proliferation within host cells (Kowalczyk et al., 2023). Overall, the LPS of *L. pneumophila*, particularly the core region and its O-acetylation status, plays a pivotal role in mediating adhesion to *A. castellanii*. This interaction is essential for the bacterium’s initial colonization and subsequent infection within the amoebal host.

The *ladC* gene, encoding a putative adenylate cyclase, is uniquely present in *L. pneumophila* but absent in other *Legionella* species, suggesting a specialized role in its pathogenesis (Newton et al., 2006). Studies indicate that *ladC* is upregulated during macrophage infection, implicating its importance in host-pathogen interactions (Newton et al., 2006). To investigate its function, researchers generated a *ladC* mutant and evaluated its infectivity in both mammalian cells and *A. castellanii*. Using differential immunofluorescence staining to distinguish adherent from intracellular bacteria, the study revealed that the *ladC* mutant exhibited significantly reduced adherence to *A. castellanii* compared to the wild-type strain. This defect was observed at early infection time points (2–12 h), indicating that *ladC* is critical for the initial contact between *L. pneumophila* and the amoebal host. Moreover, the mutant showed impaired recovery during early infection stages, suggesting that *ladC* influences both adhesion and subsequent intracellular survival (Newton et al., 2008). The adhesion and replication defects of the *ladC* mutant were fully rescued by transcomplementation with the wild-type *ladC* gene but not with a catalytically inactive variant (*ladC* N430A/R434A), demonstrating that LadC’s enzymatic activity is required for its role in infection (Newton et al., 2008). These findings establish LadC as a key mediator of *L. pneumophila*’s initial adhesion to *A. castellanii*, with its adenylate cyclase activity being indispensable for this process. The study highlights *ladC* as a virulence factor critical for early host colonization, though the precise mechanisms linking its signaling function to bacterial adhesion remain to be elucidated.

In addition to the aforementioned adhesion-related molecules in *L. pneumophila*, researchers employed ethyl methanesulfonate (EMS) mutagenesis to generate bacterial mutants with enhanced host cell entry capacity (Cirillo et al., 2000). These selected mutants demonstrated significantly increased invasion efficiency during co-culture with host cells. To identify the genetic determinants, the team constructed a genomic library from the mutant strains and introduced it into wild-type *L. pneumophila*. Through systematic transposon mutagenesis coupled with selective entry assays, two critical genetic loci (*enh1* and *enh2*) were identified. Notably, *enh1* harbored the known *rtxA* gene, while *enh2* encoded a novel factor, *EnhC*. Heterologous expression of these genes conferred the enhanced-entry phenotype to wild-type bacteria. Functional validation via an *EnhC* deletion mutant revealed markedly impaired invasion capacity, confirming its essential role in host cell penetration (Cirillo et al., 2000). The EnhC protein may facilitate bacterial invasion by interacting with host cell surface receptors or modulating intracellular signaling pathways.

Host-specific factors also play a role in *L. pneumophila* adhesion, with the process influenced by the type of infected cell (e.g., amoebae vs. macrophages). *Acanthamoeba* approaches prey via chemotaxis and random movement (at ~0.4 μm/s), creating conditions for adhesion (Schuster and Levandowsky, 1996). A 170 kDa galactose/N-acetylgalactosamine-inhibitable lectin (Gal/GalNAc) has been identified as a receptor for *L. pneumophila* adhesion to *Hartmannella vermiformis* (Venkataraman et al., 1997). Interestingly, while Gal or GalNAc sugars completely block *L. pneumophila* adhesion to *H. vermiformis*, they only mildly affect adhesion to *A. polyphaga* (Harb et al., 1998). *L. pneumophila* exhibits high affinity for mannose receptors in *A. castellanii*, particularly the α1-3-d-mannose-binding fragment and d-mannose-binding receptor (Cao et al., 1998). However, this receptor may not be genus-specific, as d-mannose blocks uptake in *A. castellanii* but not in *A. polyphaga* (Harb et al., 1998; Declerck et al., 2007a). Additionally, cycloheximide (a protein synthesis inhibitor) and cytochalasin D (a microfilament disruptor) inhibit *A. castellanii* uptake of *L. pneumophila* but not *A. polyphaga* (Harb et al., 1998; Declerck et al., 2007a). These findings highlight significant differences in adhesion mechanisms between *L. pneumophila* and different *Acanthamoeba* hosts (e.g., *A. castellanii* vs. *A. polyphaga*) (Declerck et al., 2007b; Harb et al., 1998; Declerck et al., 2005), suggesting that *L. pneumophila* employs diverse strategies to adhere to different hosts. This diversity likely reflects host cell receptor specificity, offering new insights into the complexity of *L. pneumophila*–host interactions.

### 2.2 Phagocytosis and the dot/Icm system

The trophozoites of *Acanthamoeba* utilize surface pseudopodia to ingest bacteria, yeast, algae, and organic particles via both nonspecific pinocytosis and specific phagocytosis (Diesend et al., 2017; Khan, 2006). The Phagocytosis of *L. pneumophila* by *A. castellanii* involves curling pseudopodia, a mechanism similar to that in human macrophages (Bozue and Johnson, 1996). While host-mediated phagocytosis drives bacterial uptake, the Dot/Icm (defective in organelle trafficking/intracellular multiplication) type IVB secretion system (T4SS) of *L. pneumophila* significantly enhances this process (Hilbi et al., 2001; Khelef et al., 2001). The Dot/Icm system acts as a molecular syringe injecting effector proteins encoded by highly conserved *dot/icm* genes (Berger and Isberg, 1993; Brand et al., 1994). Remarkably, approximately 10% of the *L. pneumophila* genome encodes over 300 effector proteins, many of which contain eukaryotic domains that manipulate host pathways (e.g., preventing lysosome attachment, inhibiting phagosome maturation and acidification, and protecting bacteria from degradation) (Vincent and Vogel, 2006; Berk et al., 2008; Cazalet et al., 2004; de Felipe et al., 2008; Hubber and Roy, 2010; Nora et al., 2009; Rolando and Buchrieser, 2012). However, only a few effectors have defined functions, and their roles in amoebae remain largely unexplored due to functional redundancy and characterization challenges (Supplementary Table 3).

Phagocytosis of *L. pneumophila* is closely linked to actin dynamics (Lu and Clarke, 2005). In *D. discoideum*, this process is insensitive to cytochalasin-D but sensitive to cytochalasin-A, suggesting a role beyond simple actin polymerization (Lu and Clarke, 2005; Peracino et al., 2010; Weber et al., 2006). The effector VipA directly polymerizes actin filaments and alters host trafficking (Franco et al., 2012), though it is dispensable in *A. castellanii*, indicating host-specific functions. Coronins, actin-binding proteins conserved from amoebae to mammals (Yan et al., 2005), transiently localize to phagocytic cups during early infection before rapidly disassembling (Lu and Clarke, 2005; Hayashi et al., 2008). A coronin homolog in *A. healyi (Ahcoronin)* exhibits similar behavior, colocalizing with actin and disappearing during phagocytosis (Baldo et al., 2005). These findings suggest that actin remodeling, signaling, and phagosome formation are evolutionarily conserved mechanisms in *Legionella*-amoeba interactions.

Host predation behavior further influences these interactions. The *L. pneumophila* autoinducer LAI-1 disrupts protozoan chemotaxis, promoting bacterial uptake by *A. castellanii* (Tiaden et al., 2010; Simon et al., 2015). By restricting amoeboid motility, *L. pneumophila* concentrates host feeding in bacterial-rich areas, favoring its replication (Tiaden et al., 2010). Notably, *LAI-1* is not conserved across all *Legionella* species (Burstein et al., 2016), suggesting diverse interaction strategies. Additionally, phagocytic efficiency varies among protozoa, e.g., *A. castellanii* uptakes *L. pneumophila* more efficiently than *Naegleria lovaniensis* (Declerck et al., 2005), likely due to differences in bacterial sensing, adhesion, and phagocytic machinery.

### 2.3 Inhibition of phagosome-lysosome fusion

After *Acanthamoeba* captures microorganisms, it internalizes them into acidic phagosomes filled with enzymes. Two outcomes are possible: (1) the bacteria follow the endosomal-lysosomal pathway, where internalized material is transported to early endosomes and gradually matures into phagolysosomes through fusion/fission processes, leading to the degradation of their contents; or (2) bacteria evade phago-lysosomal fusion or resist antimicrobial factors within the phagosome, thereby escaping destruction and establish a symbiotic relationship with *Acanthamoeba* (Shin and Roy, 2008). *L. pneumophila* falls into the latter category and employs a series of survival strategies to evade endocytic digestion by preventing phagolysosome formation (Horwitz, 1983b), and establish symbiosis to form a replication niche (Horwitz, 1983a). Notably, *L. pneumophila* is rapidly internalized and creates unique compartment, the *Legionella*-containing vacuole (LCV), which evades the endocytic pathway (Derré and Isberg, 2004). The LCV membrane is surrounded by vesicles derived from mitochondria and the endoplasmic reticulum (ER) (Horwitz, 1983a; Tilney et al., 2001). By utilizing ER-derived vesicles, host proteins, and mitochondria, *L. pneumophila* remodels the LCV, establishing an intracellular niche conducive to bacterial replication prior to ribosome recruitment (Tilney et al., 2001). These molecular events are highly conserved in both amoebae and macrophages.

Within 1 h of infection, the LCV is enveloped by smooth vesicles, with at least one mitochondrion closely associated with most vacuoles (Horwitz, 1983a). The mechanisms underlying mitochondrial recruitment and its benefits for *L. pneumophila* remain elusive. Studies suggest that translocated Dot/Icm effectors may mediate mitochondrial recruitment, as *dot/icm* mutants lacking a functional Type IVB Secretion System (T4BSS) fail to recruit mitochondria (Tilney et al., 2001; Berger et al., 1994; Chong et al., 2009). While certain Dot/Icm effectors [e.g., LncP (Horwitz, 1983b) and LegS2/Spl (Degtyar et al., 2009)] target mitochondria, no direct effectors involved in mitochondrial recruitment have been identified as ER-derived vesicles and mitochondria dissipate from the LCV, ribosome numbers increase significantly, promoting *L. pneumophila* replication in macrophages (Horwitz, 1983a; Tilney et al., 2001). This phenomenon extends to various hosts, including *Acanthamoeba, H. vermiformis*, *N. fowleri*, and ciliated protozoan *Balanion* (Fields et al., 1986; Newsome et al., 1985; Abu Kwaik, 1996). Additionally, in *Acanthamoeba* (e.g., *A. castellanii* and *A. polyphaga*), LCVs form ribosome-induced phagosomes (Harb et al., 1998; Bozue and Johnson, 1996; Horwitz and Maxfield, 1984; Horwitz, 1983b). Ribosome recruitment is a conserved feature in both macrophages and amoebae, with *dot/icm* mutants unable to recruit ribosomes (Tilney et al., 2001; Berger et al., 1994). Although the molecular mechanisms remain unclear, the LCV may mimic these organelles to avoid host defense systems.

**Figure 1:**
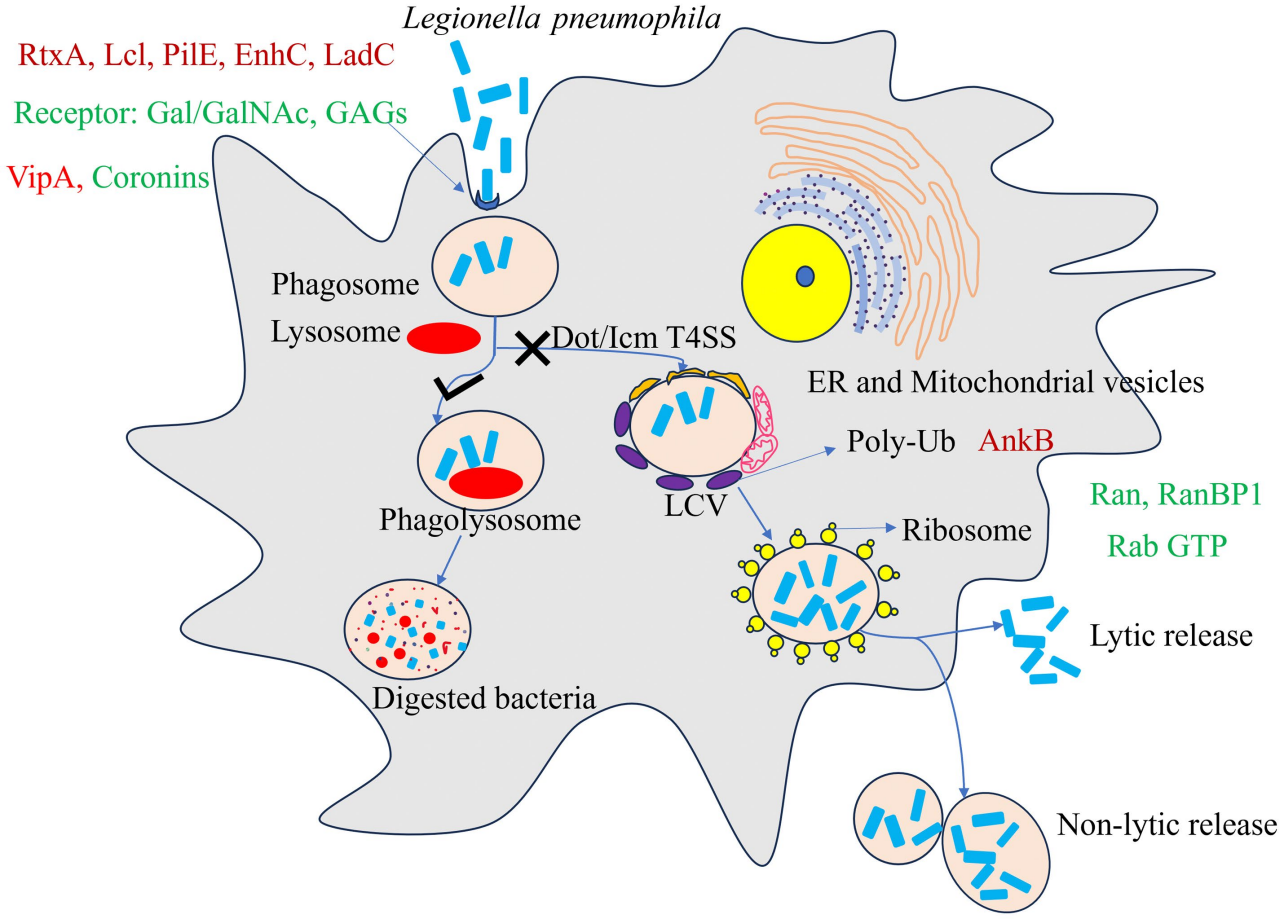
A model for Host and *Legionella pneumophila* symbiotic interactions. In non-permissive hosts, *L. pneumophila* is internalized into a phagosome, which typically fuses with lysosomes to form a degradative phagolysosome. In permissive amoebal hosts such as *Acanthamoeba* spp., *L. pneumophila* is internalized via phagocytosis and subsequently utilizes the Dot/Icm T4SS to manipulate the vacuole, forming a *Legionella*-containing vacuole (LCV) that recruits endoplasmic reticulum (ER)-derived vesicles, mitochondria, and host proteins while hijacking ribosomes to establish a replication-permissive niche. After replication, the bacteria exit via lytic and non-lytic release. The *L. pneumophila* effector AnkB activates the host ubiquitination machinery to degrade host proteins via the proteasome, releasing amino acids primarily utilized by the bacterium. Key bacterial (red) and host (green) proteins involved in this process are indicated in the figure.

A hallmark of phagosome maturation is acidification, mediated primarily by the ATP-dependent proton pump vacuolar H^+^-ATPase (v-ATPase) (Forgac, 2007). Mature phagolysosomes typically exhibit an acidic microenvironment enriched with hydrolytic enzymes capable of killing bacteria (Sturgill-Koszycki and Swanson, 2000). *L. pneumophila* targets the v-ATPase subunit VatA through its effector protein SidK, inhibiting ATP hydrolysis, proton transport, and subsequent vacuolar acidification (Xu et al., 2010). When translocated into macrophages, SidK inhibits vacuolar acidification and impairs the cell’s ability to digest non-pathogenic *Escherichia coli* (Xu et al., 2010). Studies have shown that recruitment of the v-ATPase transmembrane subunit VatM typically induces vacuolar acidification. However, in phagosomes containing wild-type *L. pneumophila*, VatM recruitment appears suppressed, preventing acidification of the LCV (Chen et al., 2004). Subsequent proteomic analyses revealed VatM in LCVs from wild-type *L. pneumophila* in PI(3)K-infected cells (Shevchuk et al., 2009; Urwyler et al., 2009). Despite these discrepancies, the absence of v-ATPase activity and its recruitment to LCVs during early infection are critical for avoiding acidification. Notably, phagosome acidification is inhibited only within the first 8 h post-infection (Horwitz and Maxfield, 1984; Horwitz, 1983a; Horwitz, 1983b). As *L. pneumophila* enters the replication phase, most infected phagosomes acquire lysosomal characteristics, and by ~18 h post-infection, vacuoles exhibit an acidic pH and endosomal markers such as lysosome-associated membrane protein 1 (LAMP-1) (Sturgill-Koszycki and Swanson, 2000). Furthermore, inhibition of LCV acidification and maturation by the v-ATPase inhibitor bafilomycin A1 effectively suppresses *L. pneumophila* replication (Sturgill-Koszycki and Swanson, 2000). The schematic diagram of the symbiotic interaction between host and *L. pneumophila* is shown in Figure 1.

### 2.4 Cellular signaling pathways

Bacterial invasion often triggers multiple host cell signaling pathways. Researchers have identified several biological pathways that may support *L. pneumophila* replication in amoebae and *A. castellanii* proliferation in natural environments. These pathways include Ran/Rab GTPase activity, tyrosine phosphorylation, mitogen-activated protein kinase (MAPK), ferrous iron transport (FeoB), and phosphoinositide (PI) signaling. Understanding these pathways may provide therapeutic targets for treatment of infection.

#### 2.4.1 Ran/Rab GTPases

Proteomic analyses have identified up to 12 small Rab GTPases involved in endosomal and vesicular trafficking as components of the LCV (Hilbi et al., 2014). Rab GTPases are central regulators of eukaryotic membrane trafficking and major targets of bacterial effectors (Brumell and Scidmore, 2007; Cossart and Roy, 2010; Stein et al., 2012; Sherwood and Roy, 2013). They play critical roles in regulating the host endocytic cycle, which is essential for nutrient uptake, immune responses, cell division, migration, and adhesion (Allgood and Neunuebel, 2018). Rab35, a key regulator, drives endocytic recycling and phagosome maturation, providing pathogens access to host resources while evading degradation (Egami et al., 2011; Verma and Datta, 2017). Although the molecular mechanisms and biological significance of bacterial exploitation of Rab GTPases remain incompletely understood, existing research highlights their precise localization and selective functions in supporting bacterial replication (Allgood and Neunuebel, 2018).

Additionally, the small GTPase Ran and its effector protein RanBP1 localize to the pathogen vacuole. Ran, which typically regulates nucleocytoplasmic transport, spindle assembly, cytokinesis, and non-centrosomal microtubule organization, is activated by the Dot/Icm substrate LegG1 in *L. pneumophila*-infected *Acanthamoeba*. LegG1 promotes microtubule stabilization through Ran activation, enhancing intracellular vacuolar motility, bacterial growth, and the chemotactic and migratory capacity of infected cells (Hilbi et al., 2014). Further studies have revealed that LegG1, like other *L. pneumophila* effectors (PpgA and PieG), contains eukaryotic RCC1 (regulator of chromosome condensation 1) repeats, which are known to activate the small GTPase Ran. Although all three effectors, LegG1, PpgA, and PieG, promote Ran activation, they target distinct components of the Ran GTPase cycle, for instance, LegG1 binds RanBP10, PpgA interacts with RanGAP1, and PieG stabilizes Ran-GTP. This functional divergence suggests that *L. pneumophila* employs a spatiotemporally regulated strategy to modulate Ran signaling during infection. The split of ancestral *pieG* into *lpg1975* and *legG1* in certain strains further highlights how evolutionary diversification of RCC1 repeat effectors fine-tunes host-pathogen interactions by expanding target specificity within the Ran GTPase network (Swart et al., 2020).

#### 2.4.2 Phosphoinositide metabolic pathway

Phosphoinositide lipid metabolism plays a pivotal role in membrane dynamics during phagocytosis, endocytosis, and exocytosis (Gillooly et al., 2001; De Matteis and Godi, 2004). Upon phagocytosis by host cells, *L. pneumophila* utilizes its Dot/Icm T4SS to deliver effector proteins that target the LCV by binding phosphoinositide lipids (Dolinsky et al., 2014). The hydrolysis of phosphoinositide (4,5)-bisphosphate (PtdIns(4,5)P2) by phosphoinositide phospholipase C (PI-PLC) generates diacylglycerol (DAG) and inositol-3-phosphate (IP3). DAG recruits C1 domain-containing proteins including protein kinase C (PKC), Rac GAP, and Rap1 to regulate phagosome maturaion (Botelho et al., 2000). Concurrently, the depletion of PtdIns(4,5)P2 is accompanied by the accumulation of DAG, PtdIns(3,4,5)P3, and PtdIns(3,4)P2, which promote actin depolymerization and facilitate phagosome remodeling (Botelho et al., 2000; Bozzaro et al., 2008; Dormann et al., 2004; Loovers et al., 2006; Scott et al., 2005; Vieira et al., 2001). These Phosphoinositides are dynamically interconverted by phosphoinositide kinases (PIKs) and phosphatases, serving as docking sites for small GTPases and proteins with specific lipid-binding domains (Lemmon, 2007; Vicinanza et al., 2008).

The class I phosphoinositide 3-kinase (PI3K) is critical for *L. pneumophila* infection, with its activity peaking during early infection stages and functioning independently of the actin cytoskeleton (Peracino et al., 2010). Interesting, while *D. discoideum* phagocytosis of *L. pneumophila* is PI3K-independent, PI3K activity inhibits bacterial replication and regulates LCV formation (Weber et al., 2006). Among the phosphoinositide-binding effectors, the SidC family plays a key role in ER recruitment to the LCV. *L. longbeachae* SidC (SidC(Llo)) exhibits a higher binding affinity for PtdIns(4)P with a Kd of 71 nM compared to its *L. pneumophila* SidC (SidC(Lpn)) (Dolinsky et al., 2014). Deletion of *sidC* impairs the recruitment of ER markers such as calnexin, Sec22b, and Rab1 to the LCV, but this defect can be functionally complemented by either SidC*Llo* or SidC*Lpn*, despite their limited sequence similarity (40%) (Dolinsky et al., 2014). These findings demonstrate that *Legionella* exploits host phosphoinositide metabolism through Dot/Icm effectors to redirect ER-derived vesicles and establish a replication-permissive niche.

#### 2.4.3 MAPK signaling pathway

MAPKs, a class of serine/threonine protein kinases, are key signaling proteins in eukaryotic cells, highly conserved from amoebae to mammals. MAPKs respond to diverse cellular stimuli, regulating gene expression, cell differentiation, proliferation, survival, and death (Shin, 2012). In mammals, the MAPK family comprises three major kinases: p38, c-Jun N-terminal kinase (JNK), and extracellular signal-regulated kinase (ERK). These kinases are activated by upstream phosphorylation events, initiating kinase cascades that phosphorylate downstream proteins (Ma and Nicolet, 2023). In *A. castellanii* and *D. discoideum*, the ERK protein family has been identified as a core component of the MAPK signaling pathway (Clarke et al., 2013; Li et al., 2009; Hadwiger and Nguyen, 2011; Aranda et al., 2025). Upon contact with *L. pneumophila*, ERK1 undergoes rapid phosphorylation (Li et al., 2009). However, whether *L. pneumophila* modulates host MAPK signaling through Dot/Icm-dependent effectors and benefits from this regulation remains unclear. Some studies suggest that *L. pneumophila* effectors inhibiting host protein synthesis may trigger MAPK activation in mouse macrophages, potentially exerting adverse effects on the bacteria (Fontana et al., 2012).

#### 2.4.4 Manipulation of host phosphorylation by bacterial phosphatases

*L. pneumophila* employs diverse effector phosphatases to hijack host tyrosine phosphorylation networks, targeting both systemic signaling pathways and localized cytoskeletal processes. The atypical HAD-like phosphotyrosine phosphatase Ceg4, translocated via the Dot/Icm system, exhibits broad phosphotyrosine dephosphorylation activity *in vitro* and suppresses MAPK signaling in yeast and human cells (Quaile et al., 2018). Through its haloacid dehalogenase (HAD) domain, Ceg4 precisely modulates the phosphorylation state of multiple MAPK cascade components (MAPKKK, MAPKK, MAPK, and MK), effectively dampening host immune responses to promote bacterial survival (Tegtmeyer et al., 2017; Krachler et al., 2011). In contrast, the classical protein tyrosine phosphatase WipA adopts a structurally distinct mechanism resembling serine/threonine phosphatases. WipA selectively dephosphorylates the Arp2/3 complex (e.g., p-N-WASP, p-ARP3) and related actin regulators (p-ACK1, p-NCK1), thereby inhibiting G-actin polymerization into F-actin and blocking phagosome maturation (Alonso and Pulido, 2016; Jia et al., 2018; Pinotsis and Waksman, 2017; He et al., 2019). This functional divergence highlights the strategy of *L. pneumophila* to concurrently disrupt global host defenses (via Ceg4-mediated MAPK inhibition) and localized barriers (via WipA-driven cytoskeletal paralysis). The evolutionary conservation of these phosphatases across bacterial strains further underscores their non-redundant roles in fine-tuning host-pathogen interactions.

#### 2.4.5 Host genetic determinants in *Legionella*-amoebae Symbiosis

The genetic characteristics of amoebae significantly influence the infection process of *L. pneumophila*. When *L. pneumophila* interacts with *H. vermiformis*, it specifically induces host cell tyrosine dephosphorylation, with the 170-kD Gal/GalNAc lectin serving as a key receptor mediating bacterial adhesion and invasion (Venkataraman et al., 1997). This lectin is expressed on the amoebal cell surface and facilitates pathogen internalization through interactions with *Legionella* effector proteins. Studies demonstrate that this dephosphorylation process also involves multiple cytoskeletal proteins, suggesting that *L. pneumophila* may promote invasion by regulating host cytoskeletal remodeling (Venkataraman et al., 1997; Venkataraman et al., 1998). Furthermore, through activation of host signaling molecules such as tyrosine phosphatases, *L. pneumophila* reshapes the intracellular environment to create favorable conditions for its survival and replication within amoebae (Venkataraman et al., 1997). These findings reveal how pathogen hijacking of host genetic background provides molecular evidence supporting the hypothesis of eukaryotic cells serving as “evolutionary crucibles.”

#### 2.4.6 Rhizoferrin transport pathway

*L. pneumophila* secretes rhizoferrin, a multicarboxylate iron carrier that facilitates growth in iron-limited environments and murine lungs. To investigate the role of the rhizoferrin biosynthesis gene (*lbtA*) in host cell infection and eliminate potential functional redundancy of the FeoB pathway, researchers constructed and analyzed *lbtA-feoB* double mutants. These mutants exhibited growth limitations in slightly iron-deficient media, confirming the central role of rhizoferrin-mediated trivalent iron uptake and FeoB-mediated ferrous iron acquisition (Lopez et al., 2023). Experiments demonstrated impaired growth of the *lbtA-feoB* mutant in *A. castellanii*, *V. vermiformis*, and human U937 macrophages, while the *lbtA*-complemented strain remained unaffected. This indicates that rhizoferrin not only regulates *L. pneumophila* extracellular survival but also plays a key role in intracellular infection. Purified rhizoferrin induced cytokine production in U937 cells, further highlighting its functional significance. Notably, rhizoferrin-related genes are highly conserved in most *L. pneumophila* strains but exhibit variability in other *Legionella* species, suggesting adaptive and survival strategy differences among strains (Lopez et al., 2023). The interplay between *L. pneumophila* and host signaling pathways is summarized in Table 1.

**Table 1:** The interaction of *Legionella pneumophila* with host cellular signaling pathways.

Signal pathway	Key proteins	Function	Effectors	Mechanism
Ran/Rab GTPases	Ran, Rab GTPases, RanBP10, RanGAP1, LegG1, PpgA, PieG, Ran-GTP	Regulation of nucleocytoplasmic transport, microtubule stabilization, vesicular trafficking	LegG1, PpgA, PieG	LegG1 binds RanBP10 and activates Ran, enhances vacuolar motility; PpgA interacts with RanGAP1 and activates Ran; PieG stabilizes microtubules
PI metabolic pathway	PI3K, PI-PLC, PtdIns(4,5)P2, DAG, IP3, SidC, calnexin, Sec22b, Arf1, Rab1	Regulation of membrane dynamics, phagosome maturation, ER recruitment	SidC, LegG1	DAG associates with actin depolymerization and phagosome maturation; PI3K inhibits bacterial replication and regulates LCV formation; SidC binds PtdIns(4)P, recruits calnexin to LCVs
MAPK signaling pathway	ERK1, MAPK, Ceg4, WipA	Regulation of gene expression, cell differentiation, proliferation, survival	Ceg4, WipA	Ceg4 inhibits MAPK signaling; WipA dephosphorylates Arp2/3 complex
Rhizoferrin transport pathway	Rhizoferrin, LbtA, FeoB	Iron acquisition in iron-limited environments	LbtA, FeoB	LbtA synthesizes rhizoferrin, FeoB transports ferrous iron; lbtA-feoB double mutants show impaired growth in iron-deficient conditions

### 2.5 The biphasic life cycle of *Legionella pneumophila* and its molecular regulatory network

*L. pneumophila* is a facultative intracellular pathogen capable of proliferating within a diverse array of host cells, including free-living protozoa, mammalian macrophages, and epithelial cells (Fields, 1996; Isberg et al., 2009). The lifecycle of this bacterium is characterized by a typical biphasic nature, comprising two key stages: the replication phase and the transmission phase (Molofsky and Swanson, 2004). During the replication phase, the bacterium employs its Dot/Icm T4SS to translocate a vast array of effector proteins into the host cell. These effectors manipulate host cell signaling and metabolic pathways to establish a conductive environment for bacterial replication. For instance, the effector protein SidC specifically recognizes PtdIns(4)P to recruit the ER around the LCVs (Dolinsky et al., 2014), while LegG1 enhances the intracellular motility of LCVs by activating the Ran GTPase (Hilbi et al., 2014). Collectively, these mechanisms create an optimal intracellular niche for bacterial proliferation.

As nutrient conditions shift and the intracellular environment no longer supports sustained bacterial growth, *L. pneumophila* transitions into the transmission phase. Transcriptomic analyses reveal that this shift is accompanied by a reprogramming of nearly half of the bacterial genes, with a significant upregulation of virulence-associated and invasive genes, particularly those encoding T4SS effectors and motility-related proteins (Brüggemann et al., 2006). This pattern of gene expression has been confirmed in both in vitro culture systems and models of infection with *A. castellanii* (Brüggemann et al., 2006).

During the transmission phase, *L. pneumophila* is released from host cells via two primary mechanisms: lytic and non-lytic release (Figure 1). In the lytic release process, the bacterium expresses pore-forming proteins such as IcmT, which create nanoscale pores in the host cell membrane, leading to osmotic lysis (Kirby et al., 1998; Gao and Kwaik, 2000). This process relies not only on the inner membrane protein encoded by the dotA gene but also on other genetic loci involved in phagosome targeting and bacterial replication (Kirby et al., 1998). Concurrently, the bacterial toxin RtxA enhances bacterial adhesion and invasiveness by binding to *β*2 integrin-like receptors on the host cell surface, thereby setting the stage for the next round of infection (Cirillo et al., 2001; Cirillo et al., 2002).

Notably, in certain *Acanthamoeba* hosts, such as *A. castellanii* and *A. polyphaga*, *L. pneumophila* can also be released in a non-lytic manner (Rowbotham, 1983; Berk et al., 1998). In this mode, intact bacterial vacuoles (typically containing 2–3 bacteria) are expelled from the host cell, which remains viable and retains its basic life functions. This unique release mechanism not only provides a potential source for the spread of Legionnaires’ disease but is also likely a crucial strategy for the bacterium’s long-term survival in environmental reservoirs such as drinking water systems. Additionally, during the transmission phase, the bacterium synthesizes the siderophore rhizoferrin to cope with iron-limited environments (Lopez et al., 2023) and fine-tunes the expression of virulence genes through non-coding RNA (ncRNA)-mediated regulation, thereby maximizing its adaptability and transmission efficiency both inside and outside the host (Romby et al., 2006).

The complex and sophisticated regulatory mechanisms of *L. pneumophila’*s lifecycle enable it to flexibly switch survival strategies across different ecological niches and host systems. By coordinating the multifaceted actions of T4SS effectors, pore-forming factors, iron acquisition systems, and gene expression regulatory networks, this pathogen not only achieves efficient host infection and intracellular proliferation but also establishes a long-term persistence capability in artificial water systems, posing a continuous challenge to public health.

### 2.6 Host benefits from the symbiosis

While much research has focused on *L. pneumophila*’s exploitation of amoebae for intracellular replication and virulence enhancement, studies demonstrate that *Acanthamoeba* can also derive benefits from this interaction. Kunze et al. (2021) employed stable isotope labeling and GC–MS metabolomics to reveal that *L. pneumophila* utilizes host amino acids for protein synthesis during intracellular growth, while concurrently enhancing the amoeba’s capacity for nutrient uptake, including improved exogenous glucose utilization via glycolysis. The AnkB effector of *L. pneumophila* activates the amoebal proteasome, elevating intracellular free amino acid concentrations (Price et al., 2011; Al-Quadan et al., 2012). These amino acids can be imported into the LCV through transporters like SLC1A5 homologs or other unidentified channels (Wieland et al., 2005; Brüggemann et al., 2006; Sauer et al., 2005; Cazalet et al., 2004; Faucher et al., 2011), indicating a metabolic cross-feeding mechanism that provides nutrients to both partners.

Beyond metabolic advantages, this symbiosis confers ecological benefits to *Acanthamoeba*. For instance, studies demonstrate that *L. pneumophila*-infected *A. polyphaga* exhibits higher resistance to certain disinfectants (e.g., NaOCl) compared to uninfected amoebae (García et al., 2007). Such reciprocal interactions establish a complex mutualism, wherein both partners gain metabolic and survival advantages, while simultaneously enhancing *L. pneumophila*’s ability to exploit host adaptations, a mechanism that further amplifies its pathogenicity, as elaborated in the subsequent section.

### 2.7 The role of *Acanthamoeba* in *Legionella pneumophila* pathogenicity

Beyond providing an intracellular niche, *Acanthamoeba* significantly enhances *L. pneumophila*’s resistance to biocides. This may result from two synergistic mechanisms: (1) *Acanthamoeba* trophozoites and cysts provide a physical barrier, reducing direct biocide contact; and (2) intracellular growth induces phenotypic changes that enhance bacterial resistance. These mechanisms are often intertwined and challenging to distinguish experimentally. Research reveals that *L. pneumophila* grown within *Acanthamoeba* exhibits distinct characteristics compared to broth-cultured bacteria. Barker et al. (1993) reported that *L. pneumophila* passaged in *A. polyphaga* displayed a surface antigen composed of a 15 kDa outer membrane protein and mono-unsaturated straight-chain fatty acids. Notably, mixing *L. pneumophila* with *Acanthamoeba* lysate did not replicate this effect. The same team found that biocides polyhexamethylene biguanide and benzisothiazolone, which severely damage the membrane integrity of broth-cultured *L. pneumophila*, showed reduced efficacy against *L. pneumophila* grown within *A. polyphaga* (Barker et al., 1992). This suggests that *Acanthamoeba*-derived proteins coating *L. pneumophila* confer biocide resistance. Additionally, *L. pneumophila* suspended in water is sensitive to 2 mg/L free chlorine (sodium hypochlorite), with viable cell numbers significantly declining after 3 min of exposure (Miyamoto et al., 2000). However, when encapsulated in *A. polyphaga* cysts, *L. pneumophila* survives for 18 h even under 50 mg/L free chlorine (Kilvington and Price, 1990), highlighting the protective role of *Acanthamoeba* cysts against biocides.

Within FLA, *L. pneumophila* forms a unique mature infectious form (MIF), morphologically distinct from bacteria grown on artificial media. MIFs are short rods with an electron-dense, layered outer membrane, containing poly-β-hydroxybutyrate granules and intramembranous layers derived from the plasma membrane (Garduño et al., 2002). Compared to fixed-phase cultures, MIFs exhibit reduced respiration rates, increased resistance to detergent-induced lysis, and enhanced survival under extreme pH conditions (Garduño et al., 2002). Furthermore, gene expression linked to intracellular infection and virulence is upregulated, bolstering *L. pneumophila*’s resistance to cationic antimicrobial peptides (Robey et al., 2001).

*Legionella pneumophila* also forms membrane-bound microporous structures within *Acanthamoeba* to evade host defenses while acquiring essential nutrients through interactions with the ER, membrane transport proteins, and cytoplasmic vesicles (Michard et al., 2015). Studies reveal that *L. pneumophila* escaping from *A. castellanii* phagosomes generates a persistent subpopulation characterized by high toxicity and antibiotic resistance (Personnic et al., 2019). These subpopulations undergo significant morphological and transcriptional changes during infection, further enhancing their adaptability and survival. Post-infection, *L. pneumophila* expresses key virulence factors, such as SdhA and LegK2, which inhibit phagosome-lysosome fusion and facilitate phagosome remodeling into replication compartments, creating favorable conditions for bacterial replication and dissemination (Gomes et al., 2018).

*Legionella pneumophila* grown within *Acanthamoeba* exhibits significantly enhanced invasiveness and pathogenicity toward human hosts. Studies demonstrate that *L. pneumophila* cultured in *A. castellanii* displays increased toxicity in a murine pneumonia model and a heightened ability to invade various cell lines, including human acute monocytic leukemia cells (THP-1), human peripheral blood mononuclear cells (hPBM), human epithelial carcinoma cells (HEp-2), and mouse leukemia monocytic macrophage cells (RAW 264.7) (Cirillo et al., 1994; Cirillo et al., 1999). Furthermore, several *Legionella* species, such as *L. gormanii*, *L. micdadei*, *Legionella steigerwaltii*, *L. longbeachae*, and *L. dumoffii*, show significantly enhanced proliferative capacity within Mono Mac 6 cells (MM6) following interaction with *A. castellanii in vitro* (Neumeister et al., 2000). These findings suggest that free-living protozoa, particularly *Acanthamoeba*, promote the expression of invasive phenotypes in *L. pneumophila* and other pathogenic microbes.

### 2.8 Potential impact on human health

*Legionella* bacteria are ubiquitous in natural water sources (e.g., rivers and lakes) and man-made water systems (e.g., cooling towers, tap water, and hot springs), with *L. pneumophila* being the most common species responsible for human disease (Kanarek et al., 2022; Carducci et al., 2010). Globally, over 10,000 cases of Legionnaires’ disease are reported annually, and *L. pneumophila* is the leading cause of waterborne disease outbreaks in the United States (van der Kooij et al., 2017; Yoder et al., 2008). A survey of 52 hot and cold water samples from urban and rural areas revealed that over 50% tested positive for *Legionella*, with detection rates of 55.88% in hot water and 55.56% in cold water (Sawczyn-Domańska, 2021). Sequencing confirmed the presence of *L. pneumophila*, and 55.17% (16/29) of positive environmental samples contained at least one virulence gene, highlighting the potential of *L. pneumophila* in water systems to cause human disease and posing a significant public health risk (Sawczyn-Domańska, 2021).

*Legionella pneumophila* can exist as free-living planktonic cells or within biofilms, adhering to filters and pipe surfaces, causing blockages, and increasing the risk of hospital-acquired infections (Mampel et al., 2006; Yoder et al., 2008; Rogers and Keevil, 1992). Lehtola et al. (2007) demonstrated that under high shear and turbulent conditions, pathogenic microorganisms such as *Mycobacterium avium*, *L. pneumophila*, *E. coli*, and cup-shaped viruses remain viable or infectious in artificial biofilms for weeks. Additionally, *L. pneumophila* can survive and grow on dead biofilm-associated microbial cells, such as those of *A. castellanii* and *Saccharomyces boulardii* (Temmerman et al., 2006). In drinking water distribution systems, biofilms may serve as persistent reservoirs for pathogenic *L. pneumophila*, exacerbating risks to human health (Shen et al., 2015).

To determine whether *L. pneumophila* in biofilms was actively growing or merely surviving in a “viable but nonculturable” state through endogenous metabolism, Murga et al. (2001) used plasmid loss as an indicator of cell division. The *L. pneumophila* strain carried a plasmid encoding kanamycin resistance and GFP, with loss of fluorescence signaling plasmid loss, and thus replication in the absence of selective pressure. When *L. pneumophila*-GFP was inoculated into a medium containing *H. vermiformis* (without a biofilm), bacterial counts increased exponentially alongside a steady decline in fluorescence. In contrast, *L. pneumophila* suspended in water without *H. vermiformis* (and without a biofilm) showed neither growth nor fluorescence loss. Biofilm reactor studies confirmed these findings, demonstrating that nearly all *L. pneumophila* cells in biofilms lacking *H. vermiformis* retained fluorescence, while those with *H. vermiformis* lost fluorescence and proliferated (Murga et al., 2001). These results suggest that while *H. vermiformis* is not essential for *L. pneumophila* survival in this system, it is required for growth.

Similar findings were reported at a international meeting (Szewzyk et al., 2000). Using a continuous flow chamber described elsewhere (Szewzyk et al., 1994), researchers tracked a *L. pneumophila* serogroup 1 strain (LP1) in biofilms and outflow water over 98 days. LP1 did not multiply in defined mixed biofilms with a natural water bacterium, and its numbers declined rapidly in outflow water. After 40 days, no LP1 cells were detected in the biofilm via *Legionella*-specific fluorescent *in situ* hybridization (FISH). In a parallel chamber where *A. castellanii* was added alongside LP1, bacterial counts in the outflow water increased by several logs and remained stable for 98 days. FISH with eukaryotic and *Legionella*-specific probes revealed colonized amoebae in the biofilm, though only half contained LP1 cells, and just 10% were heavily colonized. Some *Legionella* cells were found outside amoebae but always in close proximity. While this model system does not perfectly replicate natural drinking water biofilms, the stark contrast in *Legionella* survival and growth between biofilms with and without amoebae underscores the critical role of protozoa in sustaining *Legionella* in environmental niches.

*Legionella pneumophila* can be isolated from various environmental protozoa, a critical step in its life cycle. By parasitizing amoebae, *L. pneumophila* persists and replicates within biofilms, an adaptive survival mechanism with serious implications for human health. However, our understanding of *Legionella* concentrations in drinking water systems and its infectious dose in humans remains limited (Armstrong and Haas, 2008). Similarly, the diversity and density of protozoa that transmit *Legionella* are poorly characterized, increasing uncertainty in assessing the spread and risks of these microorganisms in water systems (Thomas et al., 2008).

Drinking water systems serve not only as potential reservoirs for pathogens but also as critical environments where biofilm-mediated growth of adapted strains occurs. Research demonstrates that *Acanthamoeba* spp. can significantly enhance the survival of various waterborne pathogens through symbiotic relationships, including *L. pneumophila*, *Mycobacterium tuberculosis*, *Helicobacter pylori*, *E. coli*, and *Mycobacterium avium* (Hojo et al., 2020; Hennebique et al., 2021; Samba-Louaka et al., 2018). These interactions not only facilitate pathogen persistence in water supply systems but may also confer chlorine resistance to bacteria (Thomas et al., 2010).

Notably, *Acanthamoeba* cysts can withstand chlorine treatment at 100 mg/L for 10 min, far exceeding conventional drinking water treatment concentrations (1–2 mg/L) (Shi et al., 2021). Significant variations exist in disinfectant susceptibility among amoebae. *Acanthamoeba* requires 3,500 mg·min /L chlorine for 4-log reduction (Loret et al., 2008), while 856 mg·min/L achieves only 2-log reduction (Dupuy et al., 2014). *Hartmannella* cysts show just 2-log reduction at 156 mg·min/L chlorine, whereas *Vermamoeba* cysts are completely inactivated by 15 mg/L chlorine for 10 min (Fouque et al., 2015). In contrast, *Naegleria* demonstrates greater sensitivity, with trophozoites inactivated by 0.79 mg/L chlorine in 30 min (Cursons et al., 1980) and cysts completely inactivated by 1.5 mg/L chlorine after 1 h (De Jonckheere and van de Voorde, 1976). However, biofilm-associated *N. fowleri* can tolerate 20 mg/L chlorine for up to 3 h (Miller et al., 2015).

Ozone treatment proves effective for water disinfection. A dose of 6.75 mg/L ozone achieves 99.9% inactivation of *Acanthamoeba* and *Naegleria* trophozoites within 30 min (Cursons et al., 1980), but shows limited efficacy against biofilm-associated *Acanthamoeba*, *Hartmannella*, and *Vahlkampfia* (Thomas et al., 2004). For UV disinfection, while WHO recommends 10 mJ/cm^2^ for 99.9% reduction of *Giardia* and *Cryptosporidium* (World Health Organization, 2004), *Acanthamoeba* trophozoites require 72.2 mJ/cm^2^ (Cervero-Aragó et al., 2014), with cysts surviving even after 800 mJ/cm^2^ exposure (Aksozek et al., 2002). *N. fowleri* needs 24 mJ/cm^2^ (trophozoites) and 121 mJ/cm^2^ (cysts) for 4-log reduction (Sarkar and Gerba, 2012). Notably, UV irradiation shows poor efficacy against biofilm-associated amoebae (Långmark et al., 2007).

These findings demonstrate the limited effectiveness of conventional disinfection methods against FLAs, particularly in cyst form. Combined treatment technologies (e.g., UV-photocatalytic oxidation) may offer more effective solutions (Ahlawat et al., 2023), though practical implementation requires careful consideration of treatment efficacy and operational feasibility.

## 3 Conclusion and future perspectives

FLAs, widely distributed protists in natural environments, serve dual ecological roles as both microbial community regulators and pathogen reservoirs. As a clinically significant FLA genus, *Acanthamoeba* spp. exhibit unique biological features: direct pathogenicity in humans (e.g., AK) and intracellular harboring of pathogens like *L. pneumophila* through endosymbiosis. This system provides an ideal model for studying microbial ecology and host-pathogen coevolution. Medically, *Acanthamoeba* functions dually as: (1) an opportunistic human pathogen, and (2) a microbial host enhancing pathogen virulence, particularly by providing protected intracellular niches (e.g., via lysosomal fusion inhibition) that increase *L. pneumophila*’s environmental resistance and pathogenic potential (Rayamajhee et al., 2021). Its environmental ubiquity makes it a critical transmission vector. Remarkably, *Acanthamoeba*’s structural and functional parallels with human macrophages establish it as a valuable surrogate model for studying pathogen-macrophage interactions (Brüssow, 2007).

The symbiotic interaction between *Acanthamoeba* and *Legionella* significantly enhances *Legionella*’s pathogenicity through three key mechanisms. First, *Acanthamoeba* provides an optimal intracellular niche for *Legionella* replication, offering abundant nutrients that facilitate high-density bacterial proliferation, thereby augmenting its pathogenic potential prior to human infection. Epidemiological evidence confirms the ubiquity of this relationship: among clinical *Acanthamoeba* strains, 61.5% (8 out of 13 strains) harbor intracellular bacteria, as demonstrated by Rayamajhee et al. (2022), a finding consistent with the 59.4% carriage rate reported in earlier studies (Iovieno et al., 2010). These bacterial communities include clinically relevant pathogens like Pseudomonas and *Legionella*, with co-infection shown to exacerbate keratitis severity (Rayamajhee et al., 2024). This interaction drives reciprocal evolutionary adaptations. *Legionella* acquires virulence genes associated with host adhesion and biofilm formation through horizontal gene transfer during its intracellular residence, while *Acanthamoeba* may derive metabolic benefits through enhanced nutrient utilization efficiency (Kunze et al., 2021; Price et al., 2011; Al-Quadan et al., 2012). Through this co-evolutionary process, *Acanthamoeba* effectively becomes a microbial “Trojan horse,” serving not only as a natural reservoir for *Legionella* but also providing a protected environment that shields pathogens from disinfectants (Shi et al., 2021) and significantly extends their environmental persistence (Bouyer et al., 2007). These combined effects ultimately amplify both the transmission risk and pathogenic potential of the harbored microorganisms.

The ecological coexistence of amoebae and *Legionella* in water systems not only reinforces their host-pathogen interaction but also poses a critical challenge to drinking water safety. *Acanthamoeba* cysts exhibit extraordinary resistance to chlorine, surviving exposures to 100 mg/L for 10 min, a concentration fifty times higher than standard water treatment doses (1–2 mg/L) (Shi et al., 2021). Current water quality assessment systems remain disproportionately focused on traditional pathogens like Giardia and Cryptosporidium (LeChevallier et al., 2024), while largely neglecting *Acanthamoeba*’s dual ecological role as both a microbial predator and potential pathogen vector. This fundamental oversight in risk assessment underscores the urgent need for a paradigm shift that integrates advances in mechanistic understanding of amoeba-pathogen interactions with the development of targeted disinfection technologies and corresponding policy reforms.

While current research has established *Acanthamoeba* as a critical driver of pathogen evolution, fundamental gaps persist across molecular, ecological, and evolutionary dimensions. Key unresolved questions include: (1) the precise mechanisms by which *L. pneumophila* hijacks host organelles (e.g., ER-mitochondria contact sites) through its effector repertoire to maintain intracellular survival while evading amoebal defenses; (2) how *Acanthamoeba*’s complex interactions with biofilm-associated microorganisms modulate *Legionella* transmission dynamics; and (3) the extent to which *Acanthamoeba* serves as a “genetic melting pot” facilitating pathogen genome innovation through horizontal gene transfer or environmental selection pressures, a process requiring validation through natural metagenomic datasets.

To comprehensively address these challenges, future research must develop integrated strategies targeting the *Acanthamoeba*-*Legionella* interaction across multiple fronts. At the molecular level, priority should be given to designing targeted inhibitors against key virulence determinants such as Dot/Icm T4SS effector proteins including SidC, alongside compounds disrupting cyst formation through chitin synthase inhibition. Concurrently, advancing monitoring capabilities will require the synergistic integration of qPCR for pathogen quantification, nanopore sequencing for strain characterization, and microfluidic platforms for single-cell behavioral analysis, collectively enabling real-time surveillance of this pathogenic partnership in water systems. For water treatment optimization, research should focus on evaluating combined disinfection approaches that leverage ultraviolet irradiation with photocatalytic oxidation technologies, while systematically assessing chlorine dosage regimens capable of overcoming the exceptional resistance exhibited by amoebal cysts. These technological advances must be paralleled by public health policy innovations, particularly the development of internationally recognized water safety standards that explicitly incorporate risk metrics for amoeba-associated pathogens, with special consideration for regions most vulnerable to climate-mediated disease transmission patterns.

Based on the aforementioned research challenges and countermeasures, the future holds promise for the establishment of a precision prevention and control system for the amoeba-pathogen symbiotic ecosystem through interdisciplinary innovation. This will provide a new solution for ensuring water safety. Breakthroughs in this field will not only deepen the understanding of host–microbe interactions but also drive the full-chain transformation from basic research to public health practice, ultimately achieving proactive defense and effective control of waterborne infectious diseases.
