# Supplementary material for: The hidden dancers in water: the symbiotic mystery of Legionella pneumophila and free-living amoebae
Source: Front Microbiol. 2025 Aug 8;16:1634806. doi: 10.3389/fmicb.2025.1634806 (PMC12370740; doi:10.3389/fmicb.2025.1634806)
Supplement: Supplementary file 1 [file Table_1.DOCX]

| **Supplementary Table 1 Amoebae that can support intracellular replication of** ***L. pneumophila*** | | | | | |
| --- | --- | --- | --- | --- | --- |
| Species | **Genus** | Class | Phylum | Type | Reference |
| *A. castellanii* | *Acanthamoeba* | Discosea | Amoebozoa | FLA | (Rowbotham, 1980; Bozue and Johnson, 1996) |
| *A. polyphaga* | *Acanthamoeba* | Discosea | Amoebozoa | FLA | (Rowbotham, 1980; Kilvington and Price, 1990; Buse and Ashbolt, 2011; Conza et al., 2013) |
| *A. hatchettii* | *Acanthamoeba* | Discosea | Amoebozoa | FLA | (Breiman et al., 1990; Conza et al., 2013; Hsu et al., 2015) |
| *A. astronyxis* | *Acanthamoeba* | Discosea | Amoebozoa | FLA | (Marciano-Cabral and Cabral, 2003; Amaro et al., 2015) |
| *A. lenticulata* | *Acanthamoeba* | Discosea | Amoebozoa | FLA | (Molmeret et al., 2001; Conza et al., 2013) |
| *A. palestinensis* | *Acanthamoeba* | Discosea | Amoebozoa | FLA | (Anand et al., 1983; Rowbotham, 1986; Harf et al., 1997) |
| *A. culbertsoni* | *Acanthamoeba* | Discosea | Amoebozoa | FLA | (Miyamoto et al., 2003) |
| *A. royreba* | *Acanthamoeba* | Discosea | Amoebozoa | FLA | (Tyndall and Domingue, 1982) |
| *A. jacobsi* | *Acanthamoeba* | Discosea | Amoebozoa | FLA | (Huang et al., 2011b) |
| *B. mandrillaris* | *Balamuthia* | Discosea | Amoebozoa | FLA | (Shadrach et al., 2005) |
| *N. australiensis* | *Naegleria* | Heterolobosea | Percolozoa | FLA | (Huang and Hsu, 2010; Conza et al., 2013) |
| *N. fowleri* | *Naegleria* | Heterolobosea | Percolozoa | FLA | (Newsome et al., 1985; Zbikowska et al., 2013; Zbikowska et al., 2014) |
| *N. gruberi* | *Naegleria* | Heterolobosea | Percolozoa | FLA | (Conza et al., 2013; Hsu et al., 2015) |
| *N. jadini* | *Naegleria* | Heterolobosea | Percolozoa | FLA | (Rowbotham, 1980) |
| *N. lovaniensis* | *Naegleria* | Heterolobosea | Percolozoa | FLA | (Declerck et al., 2007a) |
| *N. pagei* | *Naegleria* | Heterolobosea | Percolozoa; | FLA | (Huang and Hsu, 2010) |
| *C. operculata* | *Comandonia* | Heterolobosea | Percolozoa | FLA | (Breiman et al., 1990) |
| *H. cantabrigiensis* | *Hartmannella* | Tubulinea | Amoebozoa | FLA | (Breiman et al., 1990) |
| *H. vemiformisr* | *Hartmannella* | Tubulinea | Amoebozoa | FLA | (King et al., 1991) |
| *P. jugosis* | *Paratetramitus* | Heterolobosea | Percolozoa | FLA | (Breiman et al., 1990) |
| *V. ustiana* | *Vahlkampfia* | Heterolobosea | Percolozoa | FLA | (Breiman et al., 1990) |
| *D. discoideum* | *Dictyostelium* | Dictyostelia | Amoebozoa | Social amoeba | (Solomon et al., 2000) |

| **Supplementary Table 2 Epidemiological characteristics, environmental distribution, and control strategies of different L. pneumophila serogroups** | | | |
| --- | --- | --- | --- |
| Serotypes | Epidemiological characteristics | Environmental distribution, and prevention strategies | References |
| Sg 1 | - Accounts for approximately 90% of clinical cases and is strongly associated with both community-acquired and hospital-acquired Legionnaires' disease.  - Comprises multiple subtypes (e.g., Philadelphia, Knoxville, Benidorm strains), some of which exhibit enhanced virulence.  - Occasional animal infection cases have been documented. | Man-made water systems (including air conditioning units, cooling towers, water heaters, and bathwater), necessitating regular monitoring and disinfection through methods such as chlorination or copper-silver ion treatment. | (Fabbi et al., 1998; Liu et al., 1998; Yu et al., 2002; Brassinga et al., 2003; Borella et al., 2004; Thürmer et al., 2009; Amemura-Maekawa et al., 2012; Qin et al., 2014; Touray et al., 2014; Beauté, 2017; Chahin and Opal, 2017; Chaudhry et al., 2017; Petzold et al., 2017; Buse et al., 2019; Miyashita et al., 2020) |
| Sg 2-6 | - Less common, but documented cases of pathogenicity have been reported.  - Sg 2 and 3 have been reported in animal infection cases. | Predominantly colonize building water systems, with Sg 3 and 6 being more prevalent in environmental water sources, susceptibility may vary across different disinfection modalities. | (Collins et al., 1982; Fitzgeorge et al., 1983; Doleans et al., 2004; Chen et al., 2006; Fasciana et al., 2019; Natås et al., 2019; Miyashita et al., 2020; Arrigo et al., 2022; Buse et al., 2022; Scaturro et al., 2025) |
| Sg 7-15 | - Rarely associated with clinical disease.  - Sg 10 has been documented in animal infections. | **Predominantly colonize engineered water systems (e.g., cooling towers, potable water distribution systems). Sodium hypochlorite treatment combined with hyperchlorination demonstrates efficacy against Serogroup 8.** | (Edelstein et al., 1984; Thacker et al., 1986; Benson et al., 1988; Lück et al., 1994; Fabbi et al., 1998; Faris et al., 2005; Nishizuka et al., 2014; Iervolino et al., 2017; Miyashita et al., 2020; Seto et al., 2024) |

| **Supplementary Table 3 Dot/Icm-dependent translocated effectors in *Acanthamoeba/ D. discoddeum*** | | | |
| --- | --- | --- | --- |
| Effector | Amoebal model | Function | Reference |
| LegU1 | *A. castellanii* | ND | (Ensminger and Isberg, 2010) |
| Lem3 | *D. discoddeum* | ND | (Tan et al., 2011) |
| LepA | *A. castellanii* and *D. discoddeum* | Promotion of nonlytic release from protozoa | (Chen et al., 2004) |
| LepB | *A. castellanii* and *D. discoddeum* | Promotion of nonlytic release from protozoa | (Chen et al., 2004) |
| Lgt1 | *A. castellanii* | ND | (Belyi et al., 2008) |
| Lgt3 | *A. castellanii* | ND | (Belyi et al., 2008) |
| LncP | *A. castellanii* | ND | (Dolezal et al., 2012) |
| PieA | *A. castellanii* | ND | (Ninio et al., 2009) |
| SdcA | *D. discoddeum* | ER recruitment into the LCV | (Ragaz et al., 2008) |
| SdhA | *D. discoddeum* | ND | (Laguna et al., 2006) |
| SetA | *D. discoddeum* | ND | (Heidtman et al., 2009) |
| SidC | *D. discoddeum* | ER recruitment into the LCV | (Ragaz et al., 2008) |
| SidJ | *D. discoddeum* | ER recruitment into the LCV | (Liu and Luo, 2007) |
| SidK | *D. discoddeum* | ND | (Xu et al., 2010) |
| SidM/DrrA | *D. discoddeum* | Recruitment of Rab1 on the LCV membrane | (Brombacher et al., 2009) |
| VipA | *A. castellanii* | ND | (Franco et al., 2012) |
| VipD | *D. discoddeum* | ND | (VanRheenen et al., 2006) |
| LaiA/SdeA | *A. castellanii* | ND | (Bardill et al., 2005) |
| LegAU13  /AnkB/Ceg27 | *A. castellanii* and *A. polyphaga* | Acquisition of polyubiquitinated proteins by the LCV | (Lomma et al., 2010; Price et al., 2010) |
| LegC2/YIfB | *A. castellanii* and *D. discoddeum* | Alteration of normal vacuolar trafficking | (Campodonico et al., 2005; de Felipe et al., 2008) |
| LegC3 | *D. discoddeum* | Alteration of normal vacuolar trafficking | (de Felipe et al., 2008) |
| LegC7/YIfA | *A. castellanii* and *D. discoddeum* | Alteration of normal vacuolar trafficking | (Campodonico et al., 2005; de Felipe et al., 2008) |
| LegK1 | *A. castellanii* | ND | (Hervet et al., 2011) |
| LegK2 | *A. castellanii* and *D. discoddeum* | ER recruitment into the LCV | (Hervet et al., 2011) |
| LegS2 | *A. castellanii* | ND | (Degtyar et al., 2009) |
| Ceg4 | amoeba | Ceg4 is speculated to attenuates activation of eukaryotic MAPK | (Quaile et al., 2018) |
| WipA | ND | ND | (Pinotsis and Waksman, 2017) |
| LidA | *A. castellanii* | LidA binds to the protozoan Rab1 GTPase and promotes the recruitment of ER-derived vesicles to LCV | (Hoffmann et al., 2014) |
